# Viral infection in chronic otitis media with effusion in children

**DOI:** 10.3389/fped.2023.1124567

**Published:** 2023-05-10

**Authors:** Annette Runge, Sonja Straif, Zoltan Banki, Wegene Borena, Brigitte Muellauer, Juergen Brunner, Timo Gottfried, Joachim Schmutzhard, Jozsef Dudas, Brigitte Risslegger, Avneet Randhawa, Cornelia Lass-Flörl, Dorothee von Laer, Herbert Riechelmann

**Affiliations:** ^1^ Department of Otorhinolaryngology, Head and Neck Surgery, Medical University of Innsbruck, Innsbruck, Austria; ^2^ Institute of Virology, Department of Hygiene, Microbiology and Public Health, Medical University of Innsbruck, Innsbruck, Austria; ^3^ Department of Pediatrics, Medical University of Innsbruck, Innsbruck, Austria; ^4^ Faculty of Medicine and Dental Medicine, Danube Private Univeristy Krems, Krems-Stein, Austria; ^5^ Institute of Hygiene and Medical Microbiology, Medical University of Innsbruck, Innsbruck, Austria; ^6^ Department of Otolaryngology—Head and Neck Surgery, Rutgers New Jersey Medical School, Newark, United States

**Keywords:** chronic otitis media with effusion, children, respiratory virus, body mass index, immune cell exhaustion

## Abstract

**Background:**

The role of respiratory viruses in chronic otitis media with effusion (COME) in children is not clearly defined. In our study we aimed to investigate the detection of respiratory viruses in middle ear effusions (MEE) as well as the association with local bacteria, respiratory viruses in the nasopharynx and cellular immune response of children with COME.

**Methods:**

This 2017–2019 cross-sectional study included 69 children aged 2–6 undergoing myringotomy for COME. MEE and nasopharyngeal swabs were analyzed *via* PCR and CT-values for the genome and loads of typical respiratory viruses. Immune cell populations and exhaustion markers in MEE related to respiratory virus detection were studied *via* FACS. Clinical data including the BMI was correlated.

**Results:**

Respiratory viruses were detected in MEE of 44 children (64%). Rhinovirus (43%), Parainfluenzavirus (26%) and Bocavirus (10%) were detected most frequently. Average Ct values were 33.6 and 33.5 in MEE and nasopharynx, respectively. Higher detection rates correlated with elevated BMI. Monocytes were elevated in MEE (9.5 ± 7.3%/blood leucocytes). Exhaustion markers were elevated on CD4+ and CD8+ T cells and monocytes in MEE.

**Conclusion:**

Respiratory viruses are associated with pediatric COME. Elevated BMI was associated with increased rates of virus associated COME. Changes in cell proportions of innate immunity and expression of exhaustion markers may be related to chronic viral infection.

## Introduction

COME is defined as presence of fluid in the middle ear lasting longer than 3 months without signs or symptoms of acute ear infection. There are multiple risk factors, including respiratory virus infections. However, the association of respiratory viruses with other risk factors has not been clearly defined yet.

10% of all children are affected by COME after an episode of AOM, which is mostly caused by respiratory viruses (1, 2). Several respiratory viruses have been established as sources of ascending infection from the adenoids and are considered major etiological factors of COME in children (3, 4). However, those studies report variable viral detection rates in MEE of children and lack evidence of simultaneous viral occupation of the nasopharynx (5–8). Furthermore a clear separation of cases with COME from those with AOM or rAOM is missing in most reports, although onset, symptoms, clinical, microbiologic and histopathologic findings vary greatly between the diseases. In addition, no quantitative methods of genome detection were applied. Quantitative multiplex PCR for respiratory viruses is an easy, fast and undemanding tool not only for identification of the viral species, but also provides an estimate of its replicative activity and thus the time course of the infection for up to three months (9, 10).

Persistence of viral antigens might be prolonged in obese children, which is associated with reduced antiviral immunity (11). Obesity is a well- known systemic risk factor of COME and a major health concern among children in developed countries.

In addition, viral infections of the respiratory tract promote bacterial superinfections. However, the relation of viruses and otopathogenic bacteria in COME is not well established, although literature indicates that there is a larger presence of otopathogenic bacteria in children with COME than in healthy control groups (3, 6, 12).

Finally, continuous exposure of immune cells to viral antigens in the middle ear might drive immune cell exhaustion. Altered function of the immune cells could contribute to viral persistence and incomplete resolution of COME. However, apart from few studies on immune cell proliferation and infiltration from periphery in MEE, the role of immune cells in the course of COME was almost neglected until recently (13).

 This study aims to address the involvement of respiratory viruses solely in children with COME, analyze viral detection in MEE and the adenoids and the association with local bacterial occupation and the common systemic risk factor obesity. In addition, immune cell proportions and exhaustion state in COME were examined.

## Material and methods

This cross sectional prospective study was carried out between 01/2018 and 12/2019 at the Department of Otorhinolaryngology, Head and Neck Surgery at the Medical University of Innsbruck after approval from a local ethics committee (1051/2018). All research was performed in accordance with the Declaration of Helsinki. Parental written informed consent was obtained one day prior to surgery. Patients were enrolled upon outpatient visitation with indication for surgery. Patient data was collected in an excel sheet. Access to this data was limited to approved study team members.

### Study population

Patients between 2 and 6 years of age with COME and planned myringotomy and/or insertion of ventilation tubes were included in the study. Surgical intervention was indicated in patients with otoscopic evidence of refractory MEE behind an intact tympanic membrane for at least three months. Prior to surgery, conservative therapeutic measures such as Eustachian tube ventilation systems (Otovent®, Otobar®) as well as nutritive measures to lower gastroesophageal reflux had failed in those patients. Tympanometry and audiometry were performed preoperatively in all patients. The ASA Score was used to evaluate physical status of patients. Patients were included if their ASA Scores were I or II (14). Patients were excluded from the study if they had otoscopic evidence or exhibited symptoms of acute otitis media, acute upper respiratory tract infections, or congenital conditions associated with COME (trisomy 21, mucopolysaccharidosis or cleft palate). Patient history was carefully checked for known immunodeficiency, chronic cardiac or respiratory diseases. Age, sex, weight and height were recorded upon patient admittance, and BMI was classified using WHO criteria (15).

### Sample acquisition

All patients were under general anesthesia during sample acquisition. An indirect transoral nasopharyngoscopy and/or adenoidectomy, and a direct microscopic myringotomy and/or ventilation tube insertion were carried out. MEE was sampled for each ear separately using a special secretion trap (Tracheal-Saugset, Medizintechnik Andreas Fahl, Wiener Neudorf, Austria) and smeared for bacterial culturing. The adenoids were swabbed during nasopharyngoscopy. Blood samples (1.8 ml) were collected during surgery (Sarstedt S- Monovette red 1,8 ml with K3 EDTA, Sarstedt AG & Co.KG, Nümbrecht, Germany).

Samples were processed at the Institutes of Virology, Hygiene and Medical Microbiology and the Department of Dermatology at the Medical University Innsbruck within 2–24 h.

### Detection of viral genome

RNA/DNA viral extraction was performed with NucliSENS® easyMag® (bioMérieux, Vienna, Austria). Nucleic acids were extracted from 200 µl of sample to a final volume of 110 µl. In all cases, 2 µl of internal control (IC) was added directly to the lysis buffer of each extraction to check for successful extraction of the nucleic acid and monitor possible PCR inhibition. The respiratory pathogen PCR was conducted using FTD kit (Siemens Healthineers Company, Wien). This commercial kit permits the simultaneous detection of one bacterial and 19 viral pathogens, namely *Mycoplasma pneumoniae* (M.pneu), influenza virus A (INFA), influenza virus A H1N1 (INFA H1N1), influenza virus B (INFB), parainfluenzavirus 1 (PIV1), parainfluenzavirus 2 (PIV2), parainfluenzavirus 3 (PIV3), adenovirus (Ad), human metapneumovirus A and B (HMPV A/B), coronavirus NL63 (CoV-NL63), coronavirus OC43 (CoV-OC43), coronavirus 229E (CoV- 229E), coronavirus HKU1 (CoV-HKU1), RSV, RV, enterovirus (EnV),human bocavirus (hBoV), human parechovirus (hPeV) and picornavirus. A fluorescence signal from a dual-labeled probe was used to detect the presence of specific viral sequences in the reaction. Presence of viral sequences were recorded as Ct values. The limit of detection was set at >40 cycles, Ct values <30 were interpreted as active viral replication. Thermocycling was performed on a Rotor-Gene 6000/Q (QIAGEN). To validate the results, an extraction control, i.e., the IC—as well as positive and negative controls were carried out as part of the assay (16, 17).

### Culturing of bacterial smears

Culture media (Columbia bouillon, Columbia blood agar and and boiled blood agar) for aerobe cultures were inoculated with the smears and incubated over night at 37 ± 2°C for two consecutive nights. Cultures were examined for growth of pathogenes, followed by identification using MALDI TOF (MALDI TOF MS, Bruker Daltonik GmbH, Bremen, Germany). During this process pathogen-specific molecules embedded in a matrix were loosened and ionized with a laser, identified *via* mass spectrometry and compared to an online data base (18).

### Flow cytometric analysis of middle ear effusions

MEEs (500 µl–1 ml) were filled up to 10 ml with PBS supplemented with 2% FCS and 2 mM EDTA. Cell suspension was filtered through a 100 µm cell strainer to remove debris. Cell suspension was then split into four 5 ml polystyrene round-bottom tubes (both Falcon® from Corning, New York, NY, USA). Cells were pelleted by centrifugation at 1.400 rpm for 8 min at 4°C. Cell pellets were resuspended in 100 µL of PBS supplemented with 2% FCS and 2 mM EDTA. Samples were stained with either BD Multitest™ CD3/CD16 + CD56/CD45/CD19 or BD Multitest™ CD3/CD8/CD45/CD4 four-color direct immunofluorescence reagent (BD Biosciences, San Jose, CA, USA). After 30 min of incubation at 4°C samples were washed and stained with LIVE/DEAD™ fixable violet dead cell stain kit from Invitrogen (Thermo Fischer Scientific, Waltham, MA, USA). Samples were stained with BD MultitestTM CD3/CD8/CD45/CD4 with a combination of monoclonal antibodies against either PD-1 (BV510-labelled, clone EH12.1), T cell immunoglobulin and mucin-domain containing-3 (Tim-3) (BV421-labelled, clone 7D3), or Lymphocyte-activation gene 3 (Lag-3) (BV421-labelled, clone 74–530), all purchased from BD Biosciences (San Jose, CA, USA). Parallel to MEE, 100 µl of EDTA blood of the study participants was stained as described above. In blood samples, erythrocytes were lysed after the staining using BD Pharm Lyse^TM^ solution (BD Biosciences, San Jose, CA, USA). Stained cells were fixed in PBS containing 1% formalin and measured using a FACS Canto^TM^ II cell analyzer (BD Biosciences, San Jose, CA, USA). Data were analyzed with FlowJo^TM^ software (v10, BD Biosciences, San Jose, CA, USA) (13, 19). Representative data for gating and expression of check-point molecules PD-1, Tim-3 and Lag-3 are shown in Supplementary Figures S1, S2.

### Statistical analysis

Patient data, results viral genome detection and bacterial cultures were collected in an Excel sheet (Microsoft Office, 2016, Redmond, Washington, USA). Statistical analysis was performed with SPSS Statistics (IBM SPSS Statistics 25, Armonk, New York, USA). Fisher’s exact test was used to compare detection rates of viral genome. For nominal and ordinal scaled data, frequencies were recorded. Non-linear correlations of immune cell proportions and risk factors were analysed using Spearman’s Rho. A non-parametric Kruskal-Wallis test was used to determine immune cell proportions and expression of exhaustion markers PD-1, Tim-3, Lag-3 on T cells in blood and MEE.

## Results

### Population

A total of 69 children were included in the study of whom 48 (70%) were male and 21 (30%) were female. 47% of the children were below the age of 4 years. The weight of the children was classified as healthy weight (41, 59%), overweight (14, 20%) and underweight (14, 20%). Mostly symmetrical tympanograms type B and C were recorded in 67 (97%) and 2 (3%) cases, respectively. Hearing thresholds were mostly between 20 and 40 dB (34, 49%) and between 40 and 60 dB (28, 40%), a threshold between 0 and 20 dB was recorded six times (9%).

### Detection of viral genome in middle ear fluids

Viral genome was detected in MEE of 44/69 children (64%). RV had the highest prevalence (29/69, 43%), followed by PIV (18/69, 26%). RSV was not detected (Table 1). In 19/69 (27.5%) patients, the singular sample contained genome of more than one viral species genome, mostly RV and PIV (11/19 samples). If MEE existed in both ears (27 cases), separate samples of each ear were tested for viral genome. In 14/27 cases, no viral genome was detected in the second ear. Different genomes in were detected in 16/27 cases, when samples of both ears were collected. Coronaviruses were significantly more often detected in cases of bilateral MEE than in unilateral MEE (*p* = 0.048). The average Ct value was 33.6 (min 21.1, max 39.3). Ct values < 30 were measured in 10 cases (14.5%), RV (7/69, 10%), hPeV (3/69, 4%) and one simultaneous EnV. Association of virus detection in middle ear fluid with the weight was analyzed using the chi-square test. Being overweight was associated with viral presence in middle ear fluid (*p* = 0.006) (Table 2). Ct values as a measure of viral load were similar in overweight and healthy weight children (both 33.6 on avg.).

**Table 1:** Respiratory panel.

Respiratory Virus	detection in middle ear fluid (*n* = 69)	detection in the nasopharynx (*n* = 37)
Rhinovirus (RV)	29 (43%)	15 (40.5%)
Parainfluenza (PIV1,2,3)	18 (26%)	16 (43%)
Bocavirus (hBoV)	7 (10%)	6 (16%)
Coronavirus (CoV OC43, 229E, NL63, HKU1)	7 (10%)	7 (19%)
Adenovirus (Ad)	5 (7%)	8 (22%)
Parechovirus (hPeV)	2 (3%)	3 (8%)
Enterovirus (EnV)	1 (1%)	5 (13.5%)
Human Metapneumovirus (HMPV)	1 (1%)	2 (5%)
Influenza A, B. (INFA, INFA H1N1, INFB)	1 (1%)	0 (0%)
Respiratory Syncytial Virus (RSV)	0 (0%)	4 (11%)
Picornavirus	0 (0%)	1 (3%)

Percentages add up to >100% when more than one viral species was detected in a swab or MEE.

**Table 2:** Association of virus detection rate in MEE and BMI classification.

		BMI classification			Total
		Healthy weight	Above age norm	Below age norm	
Pathogenic viruses in MEE	Negative	20	1	3	24
	Positive	21	13	11	45
Total		41	14	14	69
*p* value*			.006	.07	

* Significance level *p* < .05.

### Detection of viral genome in nasopharyngeal swabs

The nasopharynx was swabbed and assessed for pathogenic viruses in 37/69 children. Positive PCR results were obtained in 31/37 patients (84%). The highest proportion of viruses detected were PIV (16/37, 43%) and RV (15/37, 40.5%), followed by Ad (8/37,22%), CoV (7/37,19%) and hBoV (6/37,16%) (Table 1). More than one (up to 5) viral species in one nasopharyngeal swab were found in 21/37 (57%) children, with RV and PIV in one third (7/21). The average Ct value was 33.5 (min 21.4, max 38.21). Ct values <30 were observed in 13 cases (35%), mostly for CoV (5/37, 13.5%), RV (4/37, 11%), and hPeV (4/37, 11%) and two simultaneous BoV and PIV. Associations of BMI and virus detection in nasopharyngeal swabs was not significant upon chi-square analysis (data not shown).

### Simultaneous detection of viral genome in MEE and nasopharyngeal swabs

Virus-PCR of middle ear fluids and nasopharyngeal swabs yielded concordant results for RVs (Cohen’s kappa = 0.4; *p* = 0.013), PIV (Cohen’s kappa = 0.55; *p* = 0.001), and hBoV (Cohen’s kappa = 0.375; *p* = 0.015). RV and PIV Results were not concordant for CoV (Cohen’s kappa =0.006; *p* = 0.97) and Ad (Cohen’s kappa = 0.1; *p* = 0.44). Ct values <30 were measured in both nasopharyngeal swabs and MEE 2 times each.

### Simultaneous detection of virus-genome and pathogenic bacteria in middle ear fluid

**Figure 1:**
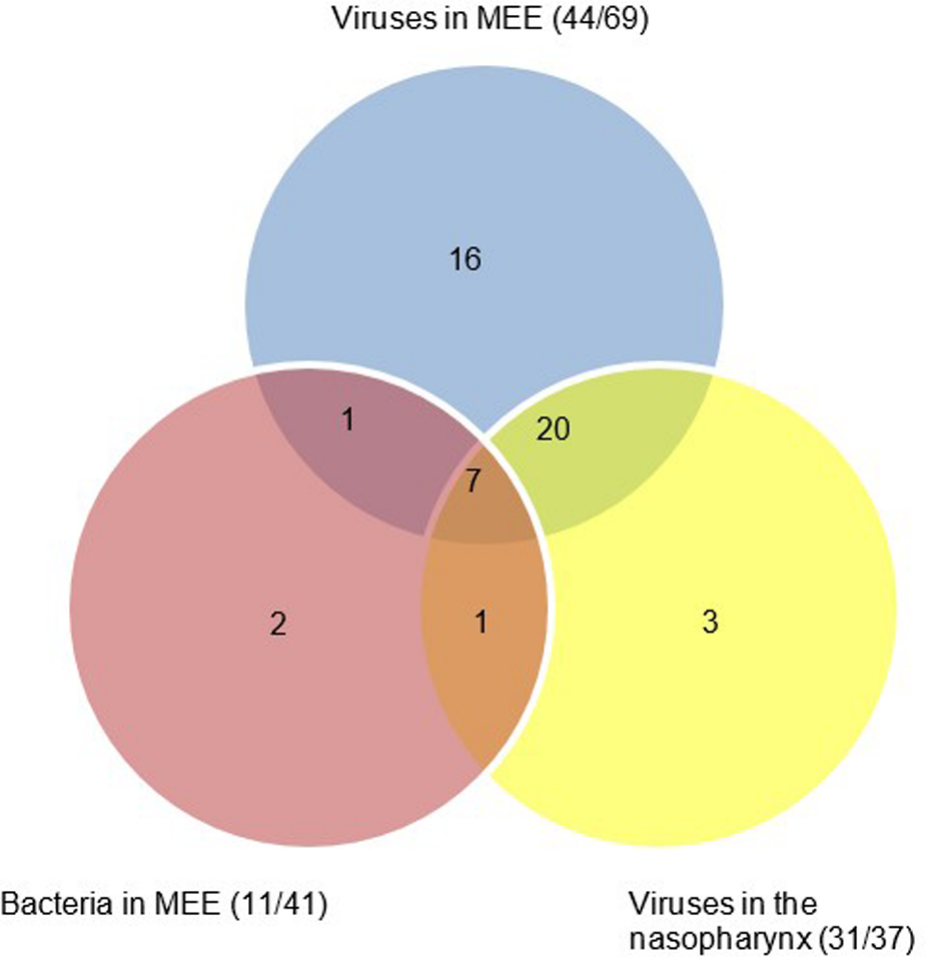
Venn diagram to illustrate joint microbial and viral occupation in the nasopharynx and middle ear fluid.

In 42/69 children bacterial smears were cultured. Positive bacterial cultures of the MEE were observed in 11 children. Haemophilus influenzae and Moraxella catarrhalis were positive in 5 children (12%) and Staphylococcus haemolyticus was positive in 1 child. Detection of bacteria in MEE was not associated with virus detection in MEE (Fisher’s exact *p* = 0.68) or virus detection in nasopharyngeal swabs (Fisher’s exact *p* = 0.61). Synchronization of numbers in a Venn diagram revealed bacterial presence in the middle ear 7 times, when viral genome was detected in MEE and the nasopharynx (Figure 1).

#### Immune cells in peripheral blood and middle ear fluid

##### Immune cells in MEE

MEE FACS was available in 39/69 children. Of the leukocytes, 23.6% were CD3+ T cells (T-lymphocytes), 10% CD4+ T cells (T- helper cells), 6.2% CD8+ T cells (cytotoxic T-cells) and 4.1% B-cells. In addition, there were 9.5% monocytes, 53.7% granulocytes and 1.8% natural killer (NK) cells (Table 3). The mean count of monocytes in MEE was slighty elevated above upper standard values.

**Table 3:** FACS analysis on immune cells in MEE in comparison to standard values in peripheral blood of children.

Immune cells	Middle Ear Fluid (% of leucocytes in MEE mean ± SD)**	Peripheral blood (% of leucocytes in peripheral blood mean ± SD)**	Normal range in peripheral blood (% of leucocytes in peripheral blood)
CD3+	23.6 ± 13.2	31.4 ± 8.9	20.1–30.1
CD4+	10.0 ± 7.0	17.4 ± 5.2	9.7–18.5
CD8+	6.2 ± 5.4	9.7 ± 3.5	3.5–13.5
B-cells	4.1 ± 3.4	8.4 ± 3.2	3.1–9.3
Monocytes	9.5 ± 7.3	6 ± 1.4	4–9
Granulocytes	53.7 ± 20.7	47.5 ± 11.6	36–77
NK-cells	1.8 ± 1.7	3.7 ± 1.9	2.3–10.4

** *N* = 39; SD, standard deviation.

Pathogen detection and immune cell proportions were correlated using Spearman’s rho tests. A positive correlation was observed for general viral detection in MEE and monocyte percentage (*p* = .04). Positive correlations were additionally observed between PIV genome detection and elevated CD8+ T cell proportions in MEE (*p* = .032) (Table 4).

**Table 4:** Association of immune cell proportions in MEE and virus detection in MEE and nasopharynx.

Immune cells	Middle Ear Fluid (% of leucocytes in MEE mean ± SD)**	Association with virus detection in MEE (*p* value after spearman’s rho testing)	Association with virus detection in nasopharynx (*p* value after spearman’s rho testing)	Association with viral species in MEE (*p* value after spearman’s rho testing)	Association with viral species in nasopharynx (*p* value after spearman’s rho testing)
CD3+	23.6 ± 13.2	.119	.341	n.s.*	n.s.
CD4+	10.0 ± 7.0	.846	.110	n.s.	**<.001 (RSV)** **^†^**
CD8+	6.2 ± 5.4	.664	.223	**.032 (PIV2)**	n.s.
B-cells	4.1 ± 3.4	.832	.722	n.s.	n.s.
Monocytes	9.5 ± 7.3	.**04**	.380	n.s.	n.s.
Granulocytes	53.7 ± 20.7	.146	.**044**	n.s.	n.s.
NK-cells	1.8 ± 1.7	.368	.636	n.s.	**.018 (RSV)**

* n.s., not significant.

** *N* = 39.

^†^ Bold numbers indicate significant levels in spearman’s rho testing. Significance level *p* < 0.05.

##### Immune cell exhaustion markers in MEE and peripheral blood

**Figure 2:**
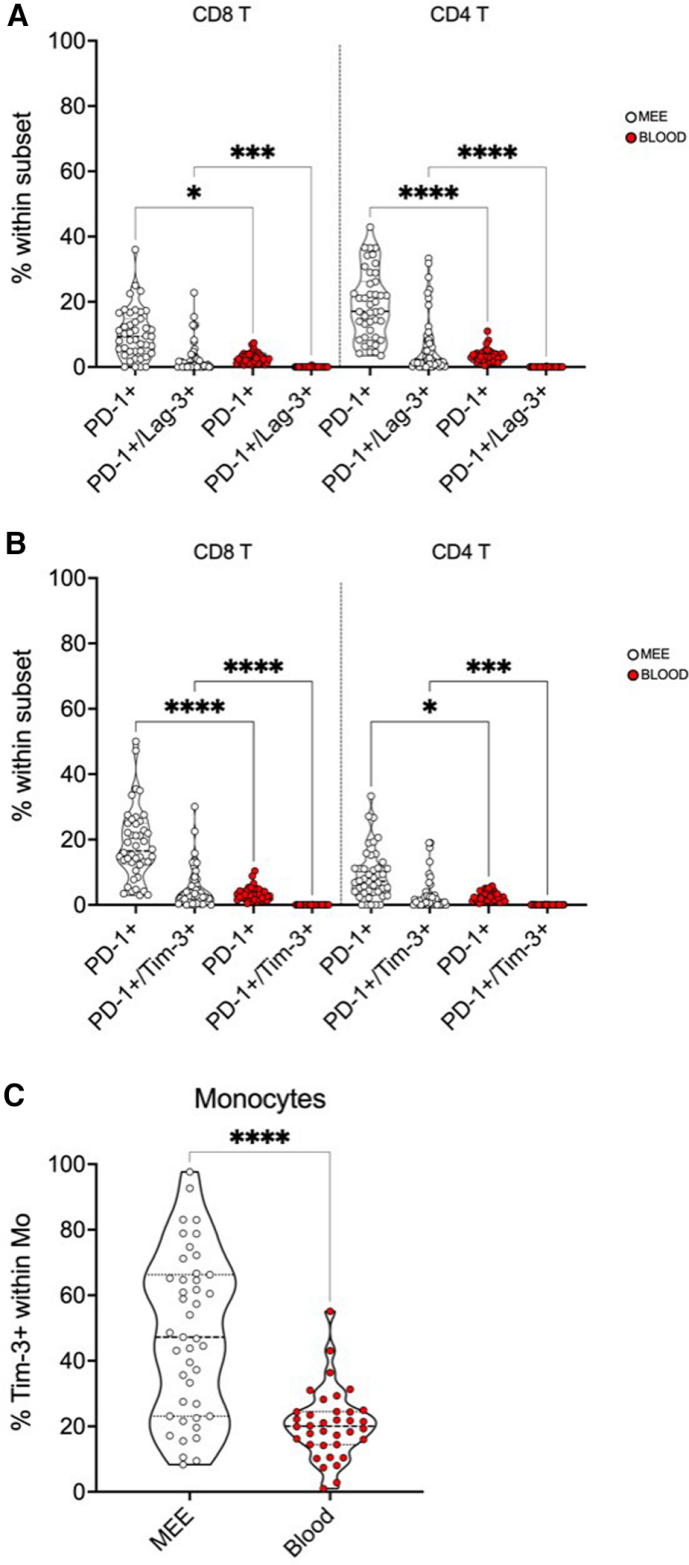
Expression of immune check-point molecules PD-1, Tim-3 and Lag-3 on immune cells in the blood and MEE. Red: immune cells in peripheral blood, white: immune cells in MEE. The number of * indicate significance levels: *p < 0.05, ***p < 0.001, ****p < 0.0001. (A) PD1/Lag-3 positive immune cells are significantly elevated in MEE in comparison with peripheral blood. (B) PD-1/Tim-3 positive immune cells are significantly elevated in MEE in comparison with peripheral blood. (C) Tim-3 positive momocytes are significantly elevated in MEE in comparison with peripheral blood.

Expression of check-point molecules PD-1, Tim-3 and Lag-3 on both CD4+ and CD8+ T cells in peripheral blood and MEE was compared (Figure 2). PD-1 positive CD4+ T cells, PD-1/Tim-3 and PD-1/Lag-3 double-positive CD4+ and CD8+ T cells and Tim-3 positive monocytes were significantly elevated in MEE compared to peripheral blood (Figures 2A–C).

## Discussion

Detection rates of respiratory viruses in MEE and adenoids of children receiving myringotomy, nasopharyngeal endoscopy and adenoidectomy for COME were addressed. Correlation with bacterial infection, viral occupation of the adenoids, abnormal BMI values and proportions and exhaustion of immune cells were analyzed.

Genomes of respiratory viruses were detected in MEE’s of 65% of the children. Similar studies found lower detection rates (56% and 52%, respectively) (6, 8). The amount of amplified genome may have been higher in our study, as other studies applied middle ear washes which may have diluted their specimens.

Genomes of RV, PIV, Ad, hBoV and CoV were most prevalent, varying roughly from 10% to 50% in MEEs. These results are in concordance with similar studies (6, 7). RSV was not detected in MEE. RSV is mostly associated with cases of symptomatic acute otitis media, which were excluded from our study (5, 20–22). Virus populations were similar in bilateral and unilateral MEE. Only coronaviruses were more often detected in cases of bilateral MEE (*p* = 0.048). However, in 5/6 cases, coronaviruses were detected simultaneously with other viruses. According to Nguyen et al., bilateral MEE was diagnosed and treated more often before the SARS CoV 2 pandemic, most likely because of more frequent office visitations and shorter waiting times for surgery appointments (23). As a result, coronaviruses should not be attributed a particular role in bilateral MEE without caution at this point.

In light of the chronic course of the disease and an exclusion from the study at symptomatic acute respiratory infection, the average Ct value of 33.5 suggested that an infection had occurred in the past and active viral replication was unlikely at the time of the study. Nucleic acids of these viral species may be shed for weeks or months after acute infections (24, 25). However, Ct values >30 may also indicate asymptomatic incubation periods. In addition, Ct values <30 were measured in 10 cases and several viral species were detected simultaneously in MEE (28%). Prolonged shedding and an overlap of infections may enhance cytopathic effects in the mucosal cells and stimulate constant release of several mucosal cytokines, resulting in inhibited mucociliary transport and a highly viscous MEE (26). The effects may be multiplied in children with above normal BMI, where more virulent mutations as well as impaired immune response to respiratory virus infections has been observed (13). These aspects certainly contribute to the known physiological stimuli of COME in overweight children, i.e., reflux and increased adipose tissue of Ostmann fatty bodies. As an elevation of the sweet and salty taste perception threshold has been shown in children with COME, the condition itself may trigger unhealthy eating habits and promote obesity (27, 28).

RVs, PIV and hBoV were most commonly simultaneously detected in the nasopharynx and in MEE. Furthermore, detection of several viral species in one sample was much more common in the nasopharynx than in MEE (58 vs. 28%). In addition, Ct values were <30 more than twice as often as in MEE (35% vs. 14.5%). Almost two thirds (20/37) of the nasopharyngeal swabs were tested positive for respiratory virus genome, when virus PCR was positive in MEE. The excess number of isolated genome detection in MEE (16/69) may be attributed to missing nasopharyngeal swabs which were not processed adequately due to various reasons. In general, these results underline the perennial reservoir function of the adenoids for respiratory viruses. According to (29), the viral load in the nasopharynx could be cleared completely by adenoidectomy.

*H. influenza* and *M. catarrhalis* were identified as the main bacterial otopathogens in this investigation. Bacterial cultures were mostly positive when viral genome (PIV and RV) was detected (Figure 1). These results suggest a viral—bacterial interaction in COME similar to observations made in the upper respiratory tract. A possible mechanism is enhanced bacterial mucosal adhererence through virally induced expression of surface proteins (i.e., ICAM 1 and PAF) in the middle ear (30, 31).

We studied proportions of immune cells in the context of respiratory virus detection in children with COME. Monocyte proportions were elevated in MEE and correlated with local detection of viral genome. In COME, monocytes have been shown to stimulate mucosal hyperplasia, vascular permeability and gas resorption in the middle ear mucosa through secretion of TNF*α*. In infants, however, monocyte functions and differentiation ability are limited (32). Elevated numbers may thus represent a compensatory mechanism for insufficient function. This assumption may be further supported by a decrease in monocyte proportions with age (*p* < 0.1).

Lymphocyte proportions in MEE were similar to those in previous studies and suggest local lymphocyte proliferation (13). Respiratory viruses have been shown to drive T cell activation directly and indirectly through stimulation of the maturation of dendritic cells and the resultant rapid lymphocyte recruitment (33). Interestingly, B and CD4+ T cells were elevated in children with BMI above the age norm in this study, possibly reflecting insufficient function of B- and T- cells in resolution of viral infection (13).

Elevated co-expression of PD-1/Lag-3 and PD-1/Tim-3 was detectable on CD4+ and CD8+ T cells. Immune cell exhaustion has been suspected to impose a “virus-host stalemate” and prevent excessive tissue damage by retaining viral replication (34). However, upregulated co- expression of inhibitory receptors and loss of antiviral effector functions have recently been associated with persistent antigen exposure, inflammation and more severe courses in COVID 19 infections (35, 36). In addition, monocytes in MEE expressed significantly more Tim-3 than those in peripheral blood. In monocytic interaction with viruses, Tim-3 inhibits pattern recognition receptors and impairs uptake of viral genome (37). Expression of exhaustion markers did not correlate with detection of viral genome or the BMI. (data not shown) These results suggest an impact of immune cell exhaustion on persistence of COME when respiratory viruses are involved. Future studies might evaluate the effect of antiviral treatment in refractory cases of COME. For clinical practice, our results underline the importance of clinical and audiometric examination of the ears in children with recurrent infections of the upper respiratory tract, as untreated COME has been shown to have a negative effect on psychosocial wellbeing of children (38). Future studies might evaluate the effect of antiviral therapy options in children with refractory cases of COME even after adenoidectomy.

### Limitations

Nasopharyngeal swabs, bacterial smears and FACS analysis were not available in all cases due to failures of sample collection and transportation. Application of viral plaque assays, confocal scan laser microscopy and tissue FAXS may enhance aspects of viral replicative activity, bacterial biofilm formation, viral- bacterial interaction and cellular composition in future experiments. In this study, pharyngeal swabs were included for the sole purpose of correlating the results with the viruses detected in the middle ear. Inclusion of additional control groups, such as children with obstructive sleep apnea undergoing adenoidectomy might broaden the knowledge on viral and bacterial profiles that trigger MEE in the future (39) In addition, we could not compare the results of MEE to control group with healthy middle ears at the same age for ethical reasons. However, the feasibility, i.e. virus detection and FACS analysis of middle ear washes, was evaluated in an adult cohort undergoing stapes surgery or cochlear implantation (see Supplementary Table S1).

## Conclusion

The results of this study suggest viral involvement in COME. Prolonged shedding and inactive viral particles in MEE and overlapping infections of the adenoids may entertain a continuous mucosal immune response. The association of an elevated BMI with respiratory virus detection underlines the immunologic impact of this risk factor. Furthermore, enhanced bacterial infection may occur in the presence of respiratory viruses in MEE. Finally, altered local cellular immune response and immune cell exhaustion exist in the presence of respiratory viruses in COME.

## Data Availability

The raw data supporting the conclusions of this article will be made available by the authors, without undue reservation.
