# Supplementary figures and images for: Viral infection in chronic otitis media with effusion in children
Source: Front Pediatr. 2023 May 10;11:1124567. doi: 10.3389/fped.2023.1124567 (PMC10208354; doi:10.3389/fped.2023.1124567)

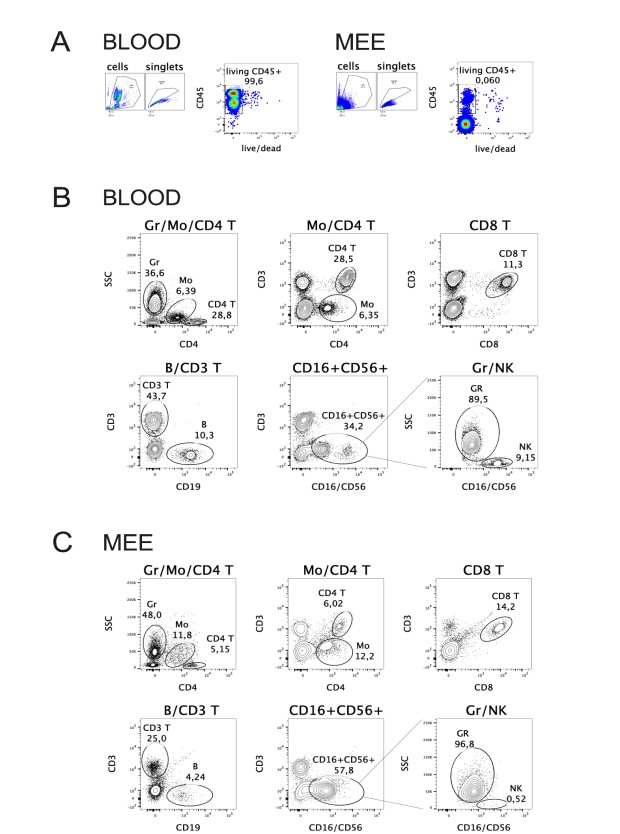

Supplement: Supplementary file 1 [file Image1.jpeg]

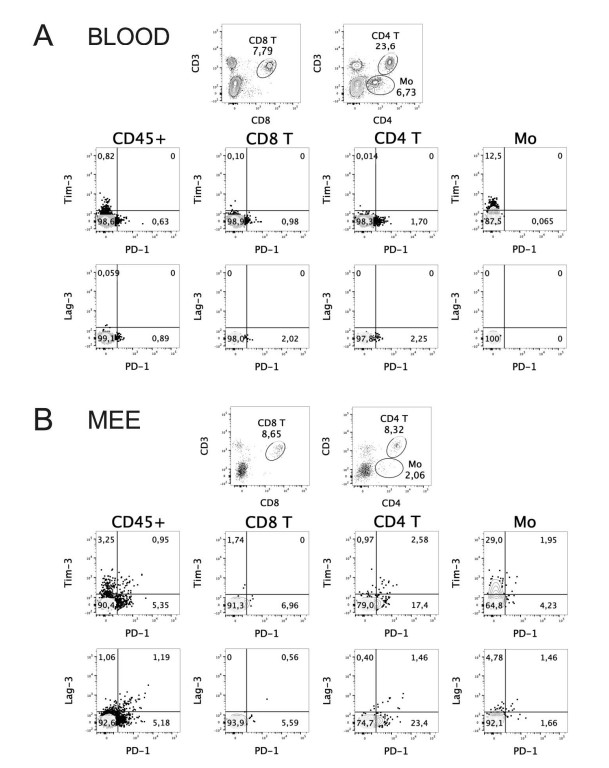

Supplement: Supplementary file 2 [file Image2.jpeg]
