# Supplementary material for: Viral infection in chronic otitis media with effusion in children
Source: Front Pediatr. 2023 May 10;11:1124567. doi: 10.3389/fped.2023.1124567 (PMC10208354; doi:10.3389/fped.2023.1124567)
Supplement: Supplementary file 3 [file Table1.docx]

| age | sex | viral genome in middle ear washes |
| --- | --- | --- |
| 74 | M |  |
| 50 | F |  |
| 60 | M |  |
| 33 | F |  |
| 57 | F |  |
| 50 | M |  |
| 71 | F |  |
| 65 | F |  |
| 25 | F |  |
| 74 | F | Parainfluenzavirus 2 |
| 30 | F |  |
| 68 | F |  |
| 81 | F | Coronavirus (OC43) |
| 58 | M | Rhinovirus, Adenovirus |
| 57 | M |  |

Suppl. Table 1

Adult patients of the control group undergoing cochlear implantation or stapes surgery. Viral genome was detected in 3 cases. M= male, F= female
